# Inferior Laryngeal Nerve Paraganglioma With Norepinephrine Hypersecretion Diagnosed Shortly After Pregnancy

**DOI:** 10.1210/jcemcr/luae107

**Published:** 2024-06-28

**Authors:** David Kishlyansky, Rithvika Ramesh, Olivia Cook, Meera Luthra

**Affiliations:** McMaster University, Division of Endocrinology and Metabolism, Hamilton, ON L8S 4L8, Canada; Michael G. DeGroote School of Medicine, McMaster University, Hamilton, ON L8P 1H6, Canada; McMaster University, Division of Endocrinology and Metabolism, Hamilton, ON L8S 4L8, Canada; McMaster University, Division of Endocrinology and Metabolism, Hamilton, ON L8S 4L8, Canada

**Keywords:** paraganglioma, pregnancy, norepinephrine, laryngeal nerve

## Abstract

The diagnosis of pheochromocytoma or paraganglioma (PGL) during pregnancy is extremely rare, with 2 large case series suggesting that the prevalence is between 0.0002% and 0.007%. Here, we present a case of a 38-year-old woman who presented during pregnancy with clinical features suggestive of preeclampsia and was found to have a norepinephrine-secreting inferior laryngeal nerve PGL, which was diagnosed after pregnancy. She underwent uncomplicated surgical resection and genetic testing revealed a succinate dehydrogenase subunit B *(SDHB)* pathogenic variant. In conclusion, PGLs diagnosed during pregnancy and hypersecreting head and neck PGLs are both rare clinical entities. Hyperfunctioning PGLs may mimic pregnancy-induced hypertension or preeclampsia. Metanephrine testing should be considered in patients with atypical features and can be reliably assessed using nonpregnant reference ranges. Overall, maternal and fetal mortality has improved considerably with early diagnosis and treatment.

## Introduction

Pheochromocytomas are rare endocrine tumors that originate from chromaffin cells in the adrenal medulla (1). When these tumors are extra-adrenal in nature, they are referred to as paragangliomas (PGL) (1). Less than 5% of all PGLs are hyperfunctioning (2). Some signs and symptoms of pheochromocytomas/PGLs include episodic or persistent hypertension, headache, diaphoresis, and palpitations (2).

Roughly 3% of PGLs are found in the head and neck (3). Head and neck PGLs (HNPGLs) have been identified in more than 20 locations, but most commonly occur in the carotid body and vagal body (4, 5). Most of these tumors are asymptomatic, solitary, and nonfunctioning (5, 6). Thus, a catecholamine-secreting neck PGL is an exceptionally rare clinical entity.

The diagnosis of pheochromocytoma or PGL during pregnancy is extremely rare, with 2 large case series suggesting that the prevalence is between 0.0002% and 0.007% (7, 8). The clinical presentation of a pregnant person with PGL is not usually different from a nonpregnant person with this condition (9). However, PGL may mimic some of the more common hypertensive disorders in pregnancy and present with similar biochemical derangements, including elevated liver enzymes, proteinuria, and thrombocytopenia (9).

Here, we present a 38-year-old woman who presented during pregnancy with clinical features suggestive of preeclampsia and was found to have a catecholamine-secreting inferior laryngeal nerve PGL.

## Case Presentation

A 38-year-old woman, gravida 1 para 0, with no medical conditions before pregnancy, presented to the hospital in April 2021 at 35 weeks and 3 days’ gestation because of malaise the preceding day with a home blood pressure reading of 160/110 mm Hg. She reported intermittent palpitations but denied headaches, diaphoresis, and pallor. There was no previous history of elevated blood pressure. She also had diet-controlled gestational diabetes and was not taking any medications, over-the-counter pills, or herbal supplements. There was no family history of endocrine disorders. She did not smoke, drink alcohol, or engage in recreational drug use.

She was assessed at labor and delivery triage, where her blood pressure was measured between 193/130 mm Hg and 210/100 mm Hg. Because of the presence of severe hypertension and elevated liver enzymes, she was diagnosed with preeclampsia (Table 1). She was treated with IV labetalol, which simultaneously acts as a nonselective antagonist of β-adrenergic receptors and a selective antagonist of α-adrenergic receptors to lower blood pressure. Despite this treatment, her hypertension became increasingly difficult to control, so she was induced, followed by an uncomplicated vaginal delivery of a healthy infant who was large for gestational age.

**Table 1.** Selected laboratory investigations from the patient's initial presentation, April 2021

Investigation	Patient value, conventional (SI)	Normal reference value, conventional (SI)
Urine ketones	Negative	Negative
Urine blood	Negative	Negative
Hemoglobin	13.6 g/dL (136 g/L)	12.0-16.0 g/dL (120-160 g/L)
Platelets	193 × 10^3^/µL (193 × 10^9^/L)	150-400 × 10^3^/µL (150-400 × 10^9^/L)
**Alanine aminotransferase**	**528 U/L (8.76 µkat/L)**	**10**-**44 U/L (0.17**-**0.73 µkat/L)**
**Alkaline phosphatase**	**271 U/L (4.53 µkat/L)**	**45**-**129 U/L (0.75**-**2.15 µkat/L)**

Bolded text indicates an abnormal result.

She re-presented to hospital on postpartum day 6 with vomiting, visual disturbances, and a home blood pressure reading of 180/90 mm Hg. In the hospital, her blood pressure was 165/89 mm Hg with persistently elevated alanine transaminase (Table 2); therefore, she was diagnosed with postpartum preeclampsia, which was treated with magnesium sulfate and 2 antihypertensives. She was discharged home on labetalol 200 mg by mouth once daily.

**Table 2.** Selected laboratory investigations from the patient's second presentation, April 2021

Investigation	Patient value, conventional (SI)	Normal reference value, conventional (SI)
Random glucose	118.9 mg/dL (6.6 mmol/L)	70.3-160.3 mg/dL (3.9-8.9 mmol/L)
**Hemoglobin**	**9.8 g/dL (98 g/L)**	**12.0**-**16.0 g/dL (120**-**160 g/L)**
Mean corpuscular volume	94 µm^3^ (94 fL)	76-96 µm^3^ (76-96 fL)
**Platelets**	**535 × 10^3^/µL (535 × 10^9^/L)**	**150**-**400 × 10^3^/µL (150**-**400 × 10^9^/L)**
**Aspartate aminotransferase**	**40 U/L (0.67 µkat/L)**	**10**-**40 U/L (0.17**–**0.67 µkat/L)**
**Alanine aminotransferase**	**136 U/L (2.23 µkat/L)**	**10**-**44 U/L (0.17-0.73 µkat/L)**
**Alkaline phosphatase**	**166 U/L (2.77 µkat/L)**	**45**-**129 U/L (0.75**-**2.15 µkat/L)**
INR	0.9	0.9-1.2
Activated partial thromboplastin time	23 s	20-28 s
**Peak troponin I (day 3)**	**646 ng/L (64.6 µg/L)**	<**17 ng/L (**<**1.7 µg/L)**

Bolded text indicates an abnormal result.

Two weeks later, her blood pressure was 179/104 and nifedipine was added. Over the next 6 months, she was weaned off both antihypertensive medications and had normal home blood pressure readings.

In May 2022 (approximately 1 year after delivery), she presented to hospital because of a 1-week history of fatigue, palpitations, and elevated blood pressure. She took an antihistamine for seasonal allergies shortly before her presentation and subsequently felt chest tightness, which lasted for a few minutes. She was not checking her blood pressure at home in the months before this. In the emergency department, she had a 30-second episode of stable supraventricular tachycardia, which resolved spontaneously. Her blood pressure was elevated at 180/100 mm Hg in both arms with a heart rate of 78 beats per minute. The physical examination was normal. There were no palpable masses or lymphadenopathy on neck examination. Her preliminary laboratory results were normal (Table 3). Her electrocardiogram showed no ischemic changes. Because of her unexplained episodes of hypertensive urgency, a secondary cause was suspected, and further endocrine workup was pursued (Tables 3 and 4). In the interim, she was treated with labetalol and nifedipine.

**Table 3.** Endocrine investigations from the patient's third hospital presentation at 14 months postpartum, June 2022

Investigation	Patient value, conventional (SI)	Normal reference value, conventional (SI)
Total urine volume	1.8 L	Nil
24-h urine creatinine	1.22 g/24 h (10.8 mmol/day)	0.62-1.98 g/24 h (5.5-17.5 mmol/day)
**24-h urine norepinephrine**	**1684 µg/24 h (9952 nmol/day)**	<**97 µg/24 h (**<**575 nmol/day)**
**24-h urine free normetanephrine**	**828 µg/24 h (4520 nmol/day)**	<**513 µg/24 h (**<**2800 nmol/day)**
24-h urine free metanephrine	21.7 µg/24 h (110 nmol/day)	<49.3 µg/24 h (<250 nmol/day)
**24-h urine free 3- methoxy tyramine**	**102.0 µg/24 h (610 nmol/day)**	<**86.9 µg/24 h (**<**520 nmol/day)**
24-h urine epinephrine	16.5 µg/24 h (90 nmol/day)	<22.0 µg/24 h (<120 nmol/day)
24-h urine dopamine	438.4 µg/24 h (2862 nmol/day)	<490.2 µg/24 h (<3200 nmol/day)
Serum aldosterone	14.3 ng/dL (395 pmol/L)	3.0-24.0 ng/dL (83-655 pmol/L)
Renin	7.9 ng/L (0.19 pmol/L)	<25.1 ng/L (0.12-0.59 pmol/L)
TSH	1.52 μIU/mL (1.52 mIU/L)	0.55-4.78 μIU/mL (0.47-4.68 mIU/L)

Bolded text indicates an abnormal result.

**Table 4.** July 22, 2022—plasma metanephrines testing at 15 months’ postpartum

Investigation	Patient value	Normal reference value
Plasma metanephrine	0.19 nmol/L	<0.5 nmol/L
Plasma normetanephrine	>7.50 nmol/L	<0.9 nmol/L
Plasma 3-methoxytyramine	0.20 nmol/L	<0.17 nmol/L

The day following her admission, she became hypotensive and both antihypertensives were held. Because of an elevated troponin level, she underwent coronary angiography, which showed possible spontaneous coronary artery dissection of her posterior interventricular artery, presumed secondary to severe hypertension with no significant coronary artery disease. She was then initiated on metoprolol 12.5 mg twice daily and discharged home with normal blood pressure readings.

## Diagnostic Assessment

**Figure 1.**
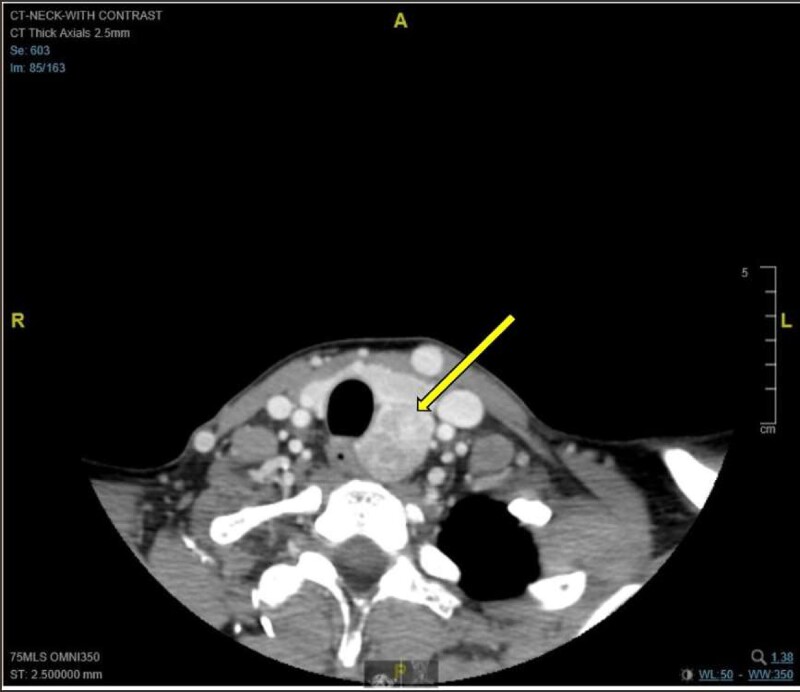
Axial CT image with contrast demonstrating a moderately avid heterogeneously enhancing exophytic mass at the posterior aspect of the left thyroid lobe, which measures 5.2 cm in the craniocaudal dimension, 2.3 cm in the mediolateral dimension, and 2.6 cm in the anteroposterior dimension. The mass extends to the left posterior paraesophageal location with mild deviation of the trachea and the esophagus to the right. There is no invasion of the surrounding structures. The remainder of the thyroid gland is normal.

A few weeks later, elevated urine norepinephrine and normetanephrine levels prompted a referral to endocrinology and metoprolol was discontinued. On confirmatory testing, her plasma normetanephrine was found to be profoundly elevated at >7.50 nmol/L (reference range, <0.90 nmol/L), consistent with a norepinephrine-secreting tumor (Table 4). An abdominal computed tomography (CT) scan demonstrated normal adrenal glands without masses. Further CT imaging of her neck and chest demonstrated an exophytic 5.2 × 2.3 × 2.6-cm mass at the posterior aspect of the left thyroid lobe, which appeared to arise from the inferior laryngeal paraganglia (Fig. 1).

**Figure 2.**
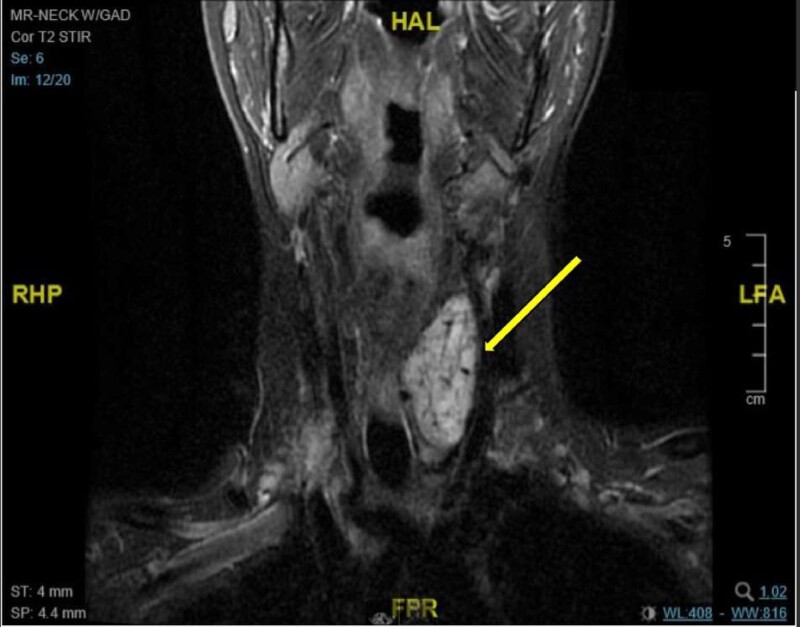
MRI scan of the neck with gadolinium (coronal MRI) demonstrating a mass in the left infrahyoid neck that is 5.6 cm in the maximal craniocaudal dimension, 2.8 cm in the anteroposterior dimension, and 2.4 cm in the mediolateral dimension. The mass is isointense to muscle on the T1-weighted sequence and demonstrates moderate T2 hyperintensity with avid postcontrast enhancement in keeping with a hypervascular mass with arterial supply that arose from the thyroid inferior mesenteric artery and left inferior thyroid artery.

After discussion at thyroid tumor board rounds, it was agreed that functional imaging (octreotide scan and/or metaiodobenzylguanidine scan) would have low sensitivity to detect multifocal disease, and the degree of norepinephrine hypersecretion was consistent with the size of this neck tumor. A magnetic resonance imaging (MRI) scan was performed to further assess the tumor and vascular anatomy, which demonstrated a 5.6 × 2.8 × 2.4-cm mass posterior to the thyroid with high suspicion of a PGL (Fig. 2). A Ga-68 DOTATATE PET CT was considered but the nearest center was 2 hours away for the patient; this was inconvenient given her young, breastfed baby, and there were booking delays because of the COVID-19 pandemic. Therefore, in discussion with the patient and surgeon, the decision was made to proceed with surgical resection based on CT and MRI findings.

## Treatment

She was prescribed doxazosin 2 mg daily, which was uptitrated to 3 mg daily, at which point she developed orthostatic hypotension. Salt tablets were prescribed, and orthostatic vital signs were monitored closely during doxazosin dose titration, with a final dose of 3 mg in the morning and 2 mg at night. This allowed her to achieve target blood pressure readings averaging less than 130 mm Hg systolic when supine and more than 90 mm Hg systolic with standing. She did not require a β-adrenergic blocker given her normal heart rate.

Because of the highly vascular nature of the mass, she underwent embolization of 2 branches of the thyrocervical trunk 1 day before her planned surgery to reduce the risk of significant bleeding. This resulted in a blood pressure drop from 142/73 to 129/76.

During surgery, the left thyroid lobe and mass were removed and large feeding vessels were clipped, which immediately brought down her heart rate and blood pressure. The recurrent laryngeal nerve entered the tumor capsule and splayed into many branches, which were too embedded into the mass to be dissected freely, so the left recurrent laryngeal nerve was sacrificed. Surgery was otherwise uncomplicated with estimated blood loss of 100 mL.

## Outcome and Follow-up

Postoperatively, her blood pressure and heart rate remained normal without any medication. Two months after surgery, plasma-free metanephrine and normetanephrine levels were normal (0.16 nmol/L [normal, <0.50 nmol/L] and 0.45 nmol/L [normal, <0.90 nmol/L], respectively) along with normal TSH and calcium. Her surgical pathology is outlined in Table 5. Genetic testing revealed a succinate dehydrogenase subunit B *(SDHB)* pathogenic variant. Given this, she will have lifelong monitoring with annual metanephrine testing and whole-body MRI every 2 to 3 years to detect any evidence of tumor recurrence, as recommended by current consensus guidelines (10).

**Table 5.** Pathology

Pathology category	Tumor characteristic	Result
Tumor location and features	Anatomic location	Left neck
	Greatest dimension	4.6 cm
	Focality	Unifocal
Histologic features	Growth pattern	Nested (alveolar, zellballen) pattern
	Composite tumor elements	Absent
	Cytologic variants	Epithelioid
	Necrosis	Not identified
	Mitotic rate	<2 mitoses (per 10 high power fields or mm^2^)
	Encapsulation	Focal capsular invasion
	Additional features	None
Invasive growth	Tumor capsule invasion	Present
	Surrounding tissues	Thyroid identified
	Lymphatic invasion	Not identified
	Surgical margins	Uninvolved
Metastases	Lymph nodes	Uninvolved (0/3 lymph nodes)
	Distant	Not applicable/not assessed
Immunochemistry stain	Chromogranin A	Positive
	GATA3	Negative
	Tyrosine hydroxylase	Positive
	S100 protein	Positive in tumor cells and strong in sustentacular cells
	Ki67	<1%
Other	Tissue	Benign thyroid tissue
		Immunostain Ki-67 shows positive staining in less than 1% of the tumor cells

## Discussion

Head and neck PGLs are rare neuroendocrine tumors originating from the neural-crest cell clusters in the head and neck and are frequently nonfunctioning (3). We present a unique case of a functional norepinephrine-secreting inferior laryngeal PGL that was diagnosed shortly after pregnancy. In one of the largest case series of patients with HNPGL (n = 54), most individuals presented with diffuse neck swelling and/or neck pain because of mass effect or cranial nerve involvement (11). Although the majority (>90%) of HNPGLs do not secrete excess catecholamines, assessment for functionality is recommended by guidelines to minimize the risk of life-threatening perioperative complications in the few patients, such as ours, who do have a hypersecretory syndrome (4). Postoperatively, in patients with functioning PGLs, metanephrine testing can be used as an adjunct for disease surveillance.

Although our patient's PGL was diagnosed shortly after pregnancy, autonomous norepinephrine hypersecretion likely contributed to her apparent preeclampsia diagnosis, as well as repeated episodes of hypertensive urgency. Older studies have suggested that unrecognized PGLs in pregnancy carries a maternal and fetal mortality rate of approximately 50% (8). Reassuringly, with early treatment, maternal and fetal morbidity and mortality is significantly reduced (12, 13). In the largest retrospective cohort study of pregnant patients (n = 232) who were diagnosed with pheochromocytoma or PGL shortly before pregnancy, during, or after delivery, unrecognized or untreated PGL was associated with a 27-fold increased risk of maternal or fetal complications (12). Complications related to untreated catecholamine hypersecretion included maternal and/or fetal death and severe cardiac events. Catecholamine levels greater than 10-fold were associated with a higher risk of adverse events (OR, 4.7; 95% CI, 1.8-13.8) and the use of antepartum α-adrenergic antagonists reduced the risk of complications (OR, 3.6; 95% CI, 1.1-13.2).

Recent literature suggests that PGLs have the highest heritability rate of any known human neoplasm, with germline mutations in driver genes identified in 40% of patients (14, 15). Therefore, guidelines now recommend genetic testing in all patients diagnosed with PGLs (4). Our patient was found to have a pathogenic variant in the *SDHB* gene. *SDHx* pathogenic variants are the most frequent genetic cause of extra-adrenal PGLs, with *SDHB* and *SDHD* pathogenic variants being the most common (15). *SDHB* pathogenic variants are associated with an earlier age of tumor onset and increased risk of tumor progression, metastases, and recurrence.

Although our patient was diagnosed with PGL after her pregnancy, a few clinical caveats are important to discuss if the diagnosis were to be considered during pregnancy.

First, pregnancy-induced hypertension (or preeclampsia) is unlikely in a patient who presents before 20 weeks of gestation or in a hypertensive patient with episodes of orthostatic hypotension (14). This should raise a concern about possible secondary causes of hypertension.

Second, plasma and urinary metanephrines are usually normal or only mildly elevated in healthy pregnant women and in those with pregnancy-induced hypertension (16). Therefore, plasma and urine metanephrines can be reliably assessed in pregnancy using nonpregnant reference ranges; elevations should raise suspicion and prompt further workup.

Third, MRI without gadolinium and ultrasound are the only imaging modalities recommended for localization of a biochemically confirmed tumor during pregnancy (14).

Last, as in nonpregnant patients, appropriate presurgical preparation with α-adrenergic antagonism is recommended in all pregnant patients with catecholamine-secreting PGLs (12). According to the Food and Drug Administration, phenoxybenzamine and other α-adrenergic blockers are currently labelled as pregnancy category C, implying the absence of data in pregnant patients (14). However, the benefits of these medications likely outweigh their potential teratogenic risks, with data demonstrating a reduction in mortality from 50% (untreated) to 5% to 15% for both mother and fetus with treatment (12). From a surgical standpoint, the current consensus is to operate during the second trimester to minimize the risks of abortion and preterm delivery in the first and third trimesters, respectively (14). For delivery in women who have not yet undergone operative management, an older systematic review (n = 89) suggested that cesarean section may result in reduced maternal mortality compared to vaginal delivery (17). However, a more recent multicenter retrospective study and literature review (n = 156) found no statistically significant difference in complications when comparing cesarean and vaginal deliveries (12).

In conclusion, PGLs diagnosed during pregnancy and hypersecreting head and neck PGLs are both rare clinical entities. Hyperfunctioning PGLs may mimic pregnancy-induced hypertension or preeclampsia, and metanephrine testing should be considered in patients with atypical features. After biochemical confirmation, imaging can be undertaken to localize the tumor and treatment with α-adrenergic blockers can be initiated safely.

## Learning Points

- All paragangliomas, regardless of location, should be investigated for excess catecholamine secretion.
- Hyperfunctioning pheochromocytomas and paragangliomas may resemble hypertensive disorders in pregnancy but atypical features, such as extreme blood pressure variability and hypertension mixed with orthostatic hypotension, should prompt investigation for a catecholamine-secreting tumor.
- In the setting of pregnancy, metanephrine testing can be reliably assessed using nonpregnant reference ranges.
- Early diagnosis and treatment of paragangliomas in pregnancy has been shown to reduce maternal and fetal morbidity and mortality.

## Data Availability

Data sharing is not applicable to this article as no datasets were generated or analyzed during the current study.

## References

[1] Neumann HPH, Young WF, Jr, Eng C. Pheochromocytoma and paraganglioma. N Engl J Med. 2019;381(6):552‐565. 31390501 10.1056/NEJMra1806651

[2] Lenders JWM, Eisenhofer G. Update on modern management of pheochromocytoma and paraganglioma. Endocrinol Metab (Seoul). 2017;32(2):152‐161. 28685506 10.3803/EnM.2017.32.2.152 PMC5503859

[3] Pellitteri PK, Rinaldo A, Myssiorek D, et al Paragangliomas of the head and neck. Oral Oncol. 2004;40(6):563‐575. 15063383 10.1016/j.oraloncology.2003.09.004

[4] Patel D, Phay JE, Yen TWF, et al Update on pheochromocytoma and paraganglioma from the SSO endocrine/head and neck disease-site work group. Part 1 of 2: advances in pathogenesis and diagnosis of pheochromocytoma and paraganglioma. Ann Surg Oncol. 2020;27(5):1329‐1337. 32112212 10.1245/s10434-020-08220-3 PMC8655649

[5] Valero C, Ganly I, Shah JP. Head and neck paragangliomas: 30-year experience. Head Neck. 2020;42(9):2486‐2495. 32427418 10.1002/hed.26277 PMC7725473

[6] Del Guercio L, Narese D, Ferrara D, Butrico L, Padricelli A, Porcellini M. Carotid and vagal body paragangliomas. Transl Med UniSa. 2013;6:11‐15. 24251239 PMC3829792

[7] Misasi G, Pancetti F, Giannini A, Simoncini T, Mannella P. Pheochromocytoma diagnosed during pregnancy: a case report. Gynecol Endocrinol. 2020;36(7):650‐653. 32314609 10.1080/09513590.2020.1754392

[8] Mannelli M, Bemporad D. Diagnosis and management of pheochromocytoma during pregnancy. J Endocrinol Invest. 2002;25(6):567‐571. 12109632 10.1007/BF03345503

[9] Gruber LM, Young WF, Jr, Bancos I. Pheochromocytoma and paraganglioma in pregnancy: a new era. Curr Cardiol Rep. 2021;23(6):60. 33961120 10.1007/s11886-021-01485-4 PMC8251512

[10] Taïeb D, Nölting S, Perrier ND, et al Management of phaeochromocytoma and paraganglioma in patients with germline SDHB pathogenic variants: an international expert consensus statement. Nat Rev Endocrinol. 2024;20(3):168‐184. 38097671 10.1038/s41574-023-00926-0

[11] Singh S, Madan R, Singh MK, Thakar A, Sharma SC. Head-and-neck paragangliomas: an overview of 54 cases operated at a tertiary care center. South Asian J Cancer. 2019;8(4):237‐240. 31807486 10.4103/sajc.sajc_339_18 PMC6852631

[12] Bancos I, Atkinson E, Eng C, Young WF, Jr, Neumann HPH; International Pheochromocytoma and Pregnancy Study Group. Maternal and fetal outcomes in phaeochromocytoma and pregnancy: a multicentre retrospective cohort study and systematic review of literature. Lancet Diabetes Endocrinol. 2021;9(1):13‐21. 33248478 10.1016/S2213-8587(20)30363-6 PMC7758862

[13] Assadipour Y, Sadowski SM, Alimchandani M, et al SDHB mutation status and tumor size but not tumor grade are important predictors of clinical outcome in pheochromocytoma and abdominal paraganglioma. Surgery. 2017;161(1):230‐239. 27839933 10.1016/j.surg.2016.05.050 PMC5164946

[14] Lenders JW . Pheochromocytoma and pregnancy: a deceptive connection. Eur J Endocrinol. 2012;166(2):143‐150. 21890650 10.1530/EJE-11-0528

[15] Nölting S, Bechmann N, Taieb D, et al Personalized management of pheochromocytoma and paraganglioma. Endocr Rev. 2022;43(2):199‐239. 34147030 10.1210/endrev/bnab019 PMC8905338

[16] Natrajan PG, McGarrigle HH, Lawrence DM, Lachelin GC. Plasma noradrenaline and Adrenaline levels in normal pregnancy and in pregnancy-induced hypertension. Br J Obstet Gynaecol. 1982;89(12):1041‐1045. 7171514 10.1111/j.1471-0528.1982.tb04661.x

[17] Schenker JG, Chowers I. Pheochromocytoma and pregnancy. Review of 89 cases. Obstet Gynecol Surv. 1971;26(11):739‐747. 5173093 10.1097/00006254-197111000-00001

